# A Qualitative Study of Psychosocial Problems among Parents of Children with Cerebral Palsy Attending Two Tertiary Care Hospitals in Western India

**DOI:** 10.1155/2014/769619

**Published:** 2014-02-20

**Authors:** Somashekhar Nimbalkar, Shyamsundar Raithatha, Rutvij Shah, Dhara Antani Panchal

**Affiliations:** ^1^Department of Pediatrics, Pramukhswami Medical College, Karamsad, Anand, Gujarat 388325, India; ^2^Central Research Services, Charutar Arogya Mandal, HM Patel Academic Center, Karamsad, Anand, Gujarat 388325, India; ^3^Department of Community Medicine, Pramukhswami Medical College, Karamsad, Anand, Gujarat 388325, India

## Abstract

*Objective.* To explore the psychosocial problems faced by the parents of children with cerebral palsy (CP) in rural and urban settings. *Design.* Qualitative research design using focus group discussions (FGDs) was used for the study. *Setting.* Two FGDs comprising one at a rural tertiary level care hospital and the other at an urban tertiary level care hospital were conducted. *Participants.* A total of thirteen parents participated in the two FGDs. *Main Outcome Measured.* Psychosocial problems experienced by the parents of children suffering from CP were measured. *Results.* The problems experienced by the mothers were associated with common themes such as disturbed social relationships, health problems, financial problems, moments of happiness, worries about future of the child, need for more support services, and lack of adequate number of trained physiotherapists. All the parents had children with problems since birth and most had approached various health care providers for a cure for their child. *Conclusions.* A wide range of psychosocial problems are experienced by the parents of children with CP. Studies like this can provide valuable information for designing a family centered care programme for children with CP.

## 1. Introduction

Cerebral palsy (CP), with a prevalence of 2.83 per 1000 children among the age group of 0 to 19 years, is one of the most common causes of disability in India [1]. A child with CP suffers from several problems such as spastic paralysis, cognitive impairment, chronic pain, speech and visual impairment, and gastrointestinal and feeding problems [2]. They also have several limitations in self-care functions such as feeding, dressing, bathing, and mobility. These limitations can result in requirements for long-term care that far exceed the usual needs of normal children [3]. The difficulties faced by children with CP result in their parents experiencing a higher level of stress [4] which has an adverse effect on their physical health and social well-being [5, 6]. Changes in healthcare systems and societal attitudes have resulted in most children staying at home in the care of family rather than in an institution. Moreover, in western countries, a greater emphasis is laid on family-centered care, wherein the focus of attention is the entire family, rather than just the child, and this has been found to be highly effective [7–11]. The family, together with service providers, is able to make informed decisions about the services and supports the child and family shall receive. In order to develop a family-centered care practice, it is imperative to understand and address the psychosocial problems experienced by the caregivers of the affected children. Several studies of this kind have been undertaken in western countries [5, 12, 13] but very few are reported in India. In a country like India there are significant differences between the environments of urban and rural areas. Our study seeks to explore the psychosocial problems experienced by the parents of children with CP. An improvement in understanding can allow us to further conduct studies in our region, to determine the extent of the problem, and develop systems to address issues that are uncovered.

## 2. Methodology

A qualitative study using focus group discussions (FGDs) was done. Two FGDs were conducted, one at a rural center involving seven participants and the second at an urban tertiary care hospital involving six participants in April 2012. The rural tertiary care hospital was Shree Krishna Hospital located in Karamsad, in Anand district in the state of Gujarat, and the urban tertiary care hospital was Kashiba Children's Hospital located in Vadodara, also in Gujarat. Ethical clearance was obtained from the institutional Human Research Ethics Committee of Shree Krishna Hospital, Karamsad. The physiotherapy units of the respective hospitals were approached and the entire study plan was discussed. The physiotherapists contacted the parents of the children with cerebral palsy coming for treatment and explained about the study. The diagnosis of cerebral palsy was based on the information contained in the medical records of the patients. All those agreeing to participate were included in the study. A round table sitting arrangement was followed during the FGDs, each of which was conducted in a separate room devoid of any disturbances. The rooms were well ventilated and had space adequate enough to accommodate all participants and researchers. Nurses were assigned the responsibility of taking care of the study participants' children for the duration of each FGD. Video recordings were done for both FGDs, and the consent for which was obtained before beginning the FGD. The FGDs were facilitated by a team, comprising one moderator (SR), one recorder (DA), one observer (SN), and a person (RS) doing the recordings. Each FGD began with an introduction by the moderator of the entire team of researchers, followed by introduction from all the participants. The moderator used an FGD guide during the focus group discussions comprising 16 questions for conducting the FGD as follows.

1. How has your life changed after having a child with disability as compared to before?
2. What problems are faced by you in day to day life in the upbringing of your child?
3. What kind of feelings do you experience for yourself or for your child? For example, depression and anxiety.
4. How is your sleep?
5. What are the health problems you suffer from at present? Are any of them a result of caring for the child?
6. What are the kinds of worries you face for the future of your child and for yourself?
7. Do you have feelings of anger or angst due to the problems faced by your child and do you blame anyone for it?
8. Can you describe moments during which you have felt happiness or satisfaction related to your child?
9. How do relatives and neighbours perceive the problems encountered by your child?
10. What is the effect of the current status of your child on your day to day work/occupation?
11. What is the effect of the current status of your child on your social relationship?
12. What is the effect of the current status of your child on your marital relationship?
13. Can you quote some instances which you might have experienced when your child has been discriminated against, in any way?
14. What is the kind of support that you have obtained from your family, general community, neighbors, school teachers, and health care providers?
15. What are financial problems experienced by you as a result of caring for your child?
16. What help do you expect and from whom in the upbringing of your child?

The questions were included in the guide based on the findings of the literature review. Moreover the FGD guide was reviewed by a paediatrician and a community medicine expert to ensure the face validity. The FGD at the rural hospital lasted for an hour and 15 minutes whereas the FGD at the urban hospital lasted for about an hour and 5 minutes. Transcripts were prepared from the audio and video recordings and were analyzed qualitatively using Atlas Ti, a textual analysis software program [14]. The data was reviewed using the software and appropriate codes were assigned to different quotations. The codes were then grouped together to create appropriate themes.

## 3. Results

All but two of the participants in the FGDs were mothers of children with CP, who were bringing their children for treatment at the respective hospitals. One of the participants at the rural hospital was the grandmother of a CP child and one at the urban hospital was the father of a CP child. There were a total of 13 participants in both FGDs combined. The sociodemographic profile of the participants is depicted in Table 1.

On analysis of the transcript of the FGDs, the following themes emerged.

*(1) Social Problems Experienced by the Parents*. One common problem reported by almost all of the participants was that their participation in social gatherings such as marriages and other ceremonies was reduced. They preferred to avoid going out during summers as they perceived going out of station to be difficult. Interactions with relatives were limited to their own homes as they could not visit relatives' houses. If the guests came to their home, attention to them was limited as priority was required to be given to child's routines such as feeding and going to sleep. Many families suffered from lack of understanding from society as a whole which resulted in adversely affected social and family relationships. One of the participants reported that if the child's father was involved in care of the child, then his peers did not approve of him. Parents were subjected to constant questioning from friends and peers, with the questions being related to duration of treatment and prognosis of the child. The questions only served to exacerbate the stress and worries of the parents. Many mothers informed that care of the child was overly time consuming and often clashed with other household duties. Upon inquiring about the effect of the child's condition that had affected their marital life, all parents maintained that it did not affect marital life significantly. One of the participants reported complaints from sibling(s) of the affected child regarding the excess attention bestowed on the affected child “You care more about my brother than me.” At those times the participant felt guilty about the disparity of attention given. There was a general opinion among participants that a balance had to be achieved between caring for affected child and the siblings.

*(2) Problems Experienced in Caring for the Child*. Many problems were experienced by the parents in caring for the child. The child kept them continuously busy. The comments made by them were “The child must stay with the mother only,” “Our baby keeps us busy at all times,” “He does not stay with anyone else so we cannot entrust him to others for even a short period of time.” The other problem reported was that they had to carry food and water everywhere they went with the child. One of the participants complained that their child was not able to tolerate heat and cried on exposure to heat. Managing the children was made more challenging as there was difficulty faced by the child in expressing their needs. The children desired playing and interacting with their peers; but as this was often not possible, parents felt that the children had negative emotions which often could not be expressed.

*(3) Financial Problems Experienced by the Parents*. It was agreed by most parents that money played an important role in the upbringing of the child. These costs ranged from the doctor's consultation and medicines to transport of the child. In some cases, as the child was more disabled, parents had to hire a separate vehicle each time in order to bring the child to and from the hospital. Many of them had to travel almost daily for obtaining physiotherapy treatment for the child. For those who ran their own business, impact was seen “I run lahri (Business on a handcart), so when I have to get the child for physiotherapy, I have to face a loss. Customers go back and business suffers.” One of the parents was a school teacher and had to leave the job in order to take care of the child. Parents brought out the point that, in order to match financial requirements for care of the child, compromises had to be made elsewhere. One of them commented that “We might eat less but we shall fulfill all that is required by him.”

*(4) Health Problems Experienced by the Parents*

*Physical Problems*. All participants unanimously reported shoulder pains and back pain because of carrying their child. Generalized body ache resulting from caring for the child and walking long distances was also reported. Parents especially experienced difficulties when taking their children to physiotherapy treatment due to lack of awareness of readily available wheelchairs. Some of them also reported sleep disturbances when the child fell ill.

*Psychological Problems Experienced by the Parents*. Parents experienced a wide variety of negative emotions which ranged from mild anger to tiredness and frustration. One of them said that at times she got very angry to the extent that she beat up her child. Another parent went to the extent of saying “At times I feel that it would have been better if i had died: so that the problems would end. The child is not developing normally, which stresses me, and increases my blood pressure, and leads to other health issues.”

*(5) Child's Future*. Most of the parents were worried about the child's future. They were concerned as who would take care of the child in their absence. Looking at the child developing and learning things, they experienced a sense of relief as it created a hope in their minds that the child would be able to learn something like how to use a computer and how to earn a livelihood. In order to overcome their worries about the child's future, they left it to destiny and were resigned to the fact that whatever is going to happen is bound to occur, and nothing is in their control.

*(6) Perception of the Society towards the Child*. Parents reported that relatives made many adverse comments about the child, which the parents found to be very disturbing. At times they felt that very few people supported them and that society was against them as a whole. They narrated experiences in which they experienced ignorance from society. A parent explained that some time back when another child of a similar age in the same colony began speaking, there was more attention given to him as compared to the participant's child. At times a direct comment was made that “Your child seems to be mentally retarded.” On accompanying the child for regular physiotherapy, a few parents reported being asked “How much longer?” by society members; the reply often was “Till we are able to make him independent.” Even the in-laws failed to understand the participants' situation and continued to have high expectations from the child. Parents responded to these situations optimistically; this included them assuming that society will continue to behave in this manner and that it would be pointless to attempt to manage their conduct and thoughts. However, instances wherein the parents were appreciated for the good care they took of their child were also reported. Some reported having very helpful and cooperative neighbors who did not misbehave with the child and family.

*(7) Response of Caregivers (Caregiver Coping Mechanisms)*. A family, in order to respond to the repeated queries of neighbors and the society, offered the explanation to others that “she (mother) works at hospital where she is taking her child for treatment and pays money for the same.” Parents also attempted to convey that caring for the child is a part of life and they have to do a bit more than others. One of the participants in the FGD was the grandmother of the affected child since the mother was staying abroad. Presence of additional family members in the home was helpful in caring for the child. One of the participant's nieces had come to stay with them, and she took care of the child in their absence. To overcome the negative feelings and thoughts about their child, they sought explanations which invoked God. One said that she felt that it would have been better if God had bestowed them a child with polio or a physically challenged child rather than such a difficult child. Another felt that God was testing them as the result of sins committed in the past. One felt that God had this destiny in store for them. One mother was confident that putting in more efforts towards the care of the child would give improved results. In this way she could ensure that her daughter would never complain that “Mummy did not do anything for my betterment.” The child's hazy future worried all of them, especially the fact about responsibility for care of the child when they would not be around. All participants shared that they felt very happy when they noticed improvement in their child such as rolling on his own, trying to walk, or attempting to vocalize a few words. At the same time they would get upset about their child when they saw other children rolling on the bed or floor or playing. Helplessness was felt by mothers when their children did not improve but the situation of the child was better now. A mother who had been looking after her child for the last 13 years commented that normal child care by itself is difficult, and caring for an affected child is even more so. Some of them were resigned to the fact that they will continue to do whatever was possible till they were alive, and that there was no point in having negative thoughts in their minds.

*(8) Support Obtained by the Caregivers from Others*. The siblings of the affected child often took over childcare in absence of parents. In one case, if the mother returned late from the hospital after receiving the treatment, the other siblings managed themselves on their own and would say “You have to take care of the little one, do not worry about us, we are fine with everything.” In case of one mother the child's grandfather took care of the child but she felt that a female was required to take care of the child in a better manner.

*(9) Support and Health Care Services*

*Health Care Services*. Most participants reported that they consulted several doctors before finally encountering the appropriate specialist. They developed a perception that there is a dearth of doctors and specialists who can and are willing to provide treatment to differently abled children. However they felt satisfied when the child demonstrated improvement after receiving treatment from the physiotherapist. The participants reported feeling disappointed when the doctor (physiotherapist) spent a lesser amount of time with the child, since they had travelled long distances and faced multiple difficulties in bringing the child to the hospital. They expected the physiotherapist to spend at least 40 minutes with the child during each session. They believed that compliance with doctor's instructions for exercise proved to be better than with the parents' instructions alone.

*Quality of Care*. Participants perceived that their child's physiotherapists were well behaved and cooperative and reported that the physiotherapists often provided emotional support as well. Participants also reported that occasional consultation on the phone was given as well.

*Response to Treatment*. Favorable improvement of the child generated commendable feedback from participants, especially since they were working very hard for the child. “We know the earlier poor condition of the child, and the improvement that we are seeing now is obvious.” “It always feels wonderful to see any new change in her. Now, things are better.” “He has become better since we started treatment here. Now he has started behaving like a normal child; he even tries to cry, eat food, walk, and so forth.”

*Support Services*. Participants reported lack of training facilities for their children. They were ready to support any institute that had educational and vocational training facilities for their children. The existing schools available for training of children with spastic CP did not admit children until the children were toilet trained. A majority of participants raised objections about the lack of special schools that would take care of their children in an appropriate manner; this was the main reason for their child not attending school. Participants also inquired about whether the hospital was going to start a special crèche, where a nurse would be available to help and teach their child, and about whether there was any other way that the hospital could help them. They wanted society to understand that by helping the affected child the entire family would be helped.

*(10) Differences between Participants Attending a Rural Clinic and Participants Attending an Urban Clinic*. The participants attending the rural hospital were more at ease and disclosed their views more openly as compared to their urban counterparts who were relatively reserved. Moreover, the former had more expectations from their care provider as compared to the latter.

## 4. Discussion

A wide range of psychosocial problems was uncovered during the focus group discussions. The participants experienced mixed responses from society; while in some cases the society neglected the child, raised several questions, and made adverse comments, in other cases neighbors provided excellent support to parents in caring for the child. In a qualitative study conducted by Neely-Barnes et al. with parents of children with autism, CP, sickle cell disease, and Down's syndrome, several instances of discrimination from society and family were reported, towards both the child and the parent [15]. Caring for the child affected the participants' relations with family members, relatives, neighbors, and others since they had to remain continuously engaged with the child. Such difficulty in maintaining social relationships was also reported in a study by Davis et al. conducted in Melbourne in 2009 [5]. One common concern experienced by all of the participants stressed on the future of the child. A similar finding was observed in the study by Heaman, in which the most common stressor reported by mothers and fathers was a concern about the child's future [16]. The participants experienced various psychological problems, such as sleep disturbances, emotional problems, and, in one of the cases, suicidal tendencies as a result of stress from caring for the child while observing no improvement in the child's status. A study conducted in Iran in 2009 has shown that having a child with CP is associated with higher prevalence and severity of depression in mothers [17]. In a study undertaken in the UK, it was found that parents of children with intellectual disabilities attained high depression and anxiety scores and that a majority met the criteria for possible clinical depression and/or anxiety, as compared to parents of normally developing children [18]. The participants in this study frequently reported back pain and generalised body aches from carrying their child to the treatment facilities. In a study conducted in Canada, the primary caregivers of children with CP reported an increased likelihood for numerous physical health problems, back pain in particular [19]. In the study by Lach et al., back pain was found to be the second most commonly reported complaint by caregivers of children with neurodevelopmental disorders [20]. Poor sleep quality was found in parents caring for children with developmental disabilities in a study conducted in England [21]. Even the smallest sign of improvement in the child gave participants immense hope, along with a sense of satisfaction. Some of the participants had to leave their jobs as a result of having to care for the child. This caused a great financial burden for them. Many of the participants experienced psychological problems as well. The general consensus among participants was that the availability of health care professionals, and other support services, for children affected with CP was inadequate. In a study conducted in Melbourne by Davis et al. in 2009 [5], similar problems were reported, in which the mothers complained of physical problems that worsened as the child grew older and heavier, for example, disturbed sleep, difficulty in maintaining social relationships, negative effect on mother's employment status, financial pressure on parents, and insufficient support services. In the same study an adverse effect on marital relationship was reported, but this was not the case in present study. The participants in the FGD undertaken at the urban-based hospital appeared to be poorly adjusted to the problems of their children as compared to the participants in the rural-based hospital. Possible explanations for this could be greater availability of social support in rural areas, greater levels of stress in the urban life style, higher cost of living in an urban setup, and so forth.

### 4.1. Limitations of the Study

The findings of the study are applicable only to a population of parents bringing their children for physiotherapy treatment at tertiary care hospitals. The scenario of parents not going for physiotherapy treatment at a smaller physiotherapy set up/facility, or of parents not going at all for treatment, may be slightly different. There are also limitations to the focus group design that would also be present. In order to effectively manage children with CP, our health care systems and other support systems should be designed in a way that the problems experienced by the parents as identified in our study are appropriately addressed.

## 5. Conclusion

Present study sheds light on the wide range of social, psychological, and health problems experienced by the parents of children with CP. A wide range of these problems are experienced by the caretakers of children with CP. While planning a family-centered program for such children, these problems should be considered and addressed in order to make care of the child more effective.

## Figures and Tables

**Table 1:** Sociodemographic characteristics of the study participants and the affected ones.

Item	Rural hospital	Urban hospital
Study participant		
Number of participants	7	6
Mean age in years	35.85	35
Mean years of schooling	10.14	6
Occupation		
Housewives	6	5
Working	1	1
Type of family		
Joint	3	4
Nuclear	4	2
Mean number of family members	6	6.17
Mean number of children	1.86	1.5
Children of the participants		
Mean age of the children in years	4.57	6.83
Sex		
Male	6	5
Female	1	1
Mean age of the child in years at the time of diagnosis	1.46	0.71 (around 9 months)
Mean duration of illness in years	3.07	6.13
